# Transcription of Muscle Actin Genes by a Nuclear Form of Mitochondrial RNA Polymerase

**DOI:** 10.1371/journal.pone.0022583

**Published:** 2011-07-25

**Authors:** Yu-Ling Lee, Chun-Hui Chiao, Ming-Ta Hsu

**Affiliations:** 1 Institute of Biochemistry and Molecular Biology, School of Life Science, National Yang-Ming University, Taipei, Taiwan; 2 VGH Yang-Ming Genome Research Center, National Yang-Ming University, Taipei, Taiwan; George Mason University, United States of America

## Abstract

Actins are the major constituent of the cytoskeleton. In this report we present several lines of evidence that muscle actin genes are transcribed by nuclear isoform of mitochondrial RNA polymerase (spRNAP-IV) whereas the non-muscle actin genes are transcribed by the conventional RNA polymerase II (PolII). We show that mRNA level of muscle actin genes are resistant to PolII inhibitors α-amanitin and triptolide as well as insensitive to knockdown of PolII but not to knockdown of spRNAP-IV, in contrast to non-muscle actin genes in several cell lines. Similar results are obtained from nuclear run-on experiments. Reporter assay using muscle actin or PolII gene promoters also demonstrate the differential sensitivity to PolII inhibitors. Finally, chromatin-immunoprecipitation experiment was used to demonstrate that spRNAP-IV is associated with promoter of muscle actin genes but not with that of non-muscle gene and knockdown of spRNAP-IV depleted this polymerase from muscle actin genes. In summary, these experiments indicate that the two types of actin genes are transcribed by different transcription machinery. We also found that *POLRMT* gene is transcribed by spRNAP-IV, and actin genes are sensitive to oligomycin, suggesting a transcription coupling between mitochondria and nucleus.

## Introduction

Transcription of mRNA in eukaryotes is generally performed by RNA polymerase II (PolII), one of the three nuclear RNA polymerases. Kravchenko *et al*., however, reported in 2005 that a fourth nuclear RNA polymerase, spRNAP-IV, also participated in the transcription of some of the nuclear mRNAs of HeLa cells [1]. spRNAP-IV and the mitochondria-targeting RNA polymerase mtRNAP are both single-polypeptide enzymes encoded by the *POLRMT* gene by differential splicing, but the former lacks the N-terminal 262-amino acids of the latter, and thus the mitochondrial targeting signal. The nature of genes transcribed by spRNAP-IV remains largely unknown. Furthermore, in the previous study the involvement of spRNAP-IV in nuclear mRNA transcription was largely based on a comparison of mRNA levels in cells treated and untreated with a PolII inhibitor α-amanitin. Because levels of specific mRNAs can also be affected by RNA processing and turnover, there is considerable uncertainty whether the observed differences were indeed due to transcription by the α-amanitin-resistant spRNAP-IV.

During our transcriptome analysis of MCF-7 cells using microarray, we observed that there were over 800 genes resistant to both PolII inhibitors, α-amanitin and triptolide [2], [3], and up-regulated more than two folds in terms of mRNA expression level. However, only one third of these genes were actually found to be insensitive to PolII knockdown and down-regulated by knockdown of spRNAP-IV in microarray analyses. To test which genes out of this subset of human genes are truly transcribed by spRNAP-IV but not by PolII, we chose a few genes for further investigation using nuclear run-on and knockdown to rule out RNA stability and processing. Among these genes we noticed that muscle actins as a group, in contrast to non-muscle actin genes, are resistant to both inhibitors of PolII and PolII knockdown as well as sensitive to spRNAP-IV knockdown. Here we present our study of the transcription of the actin group of genes, and show that those encoding for smooth, cardiac and skeletal actins are indeed transcribed by spRNAP-IV, but those non-muscle actins are transcribed by PolII. We also found that the nuclear *POLRMT* gene itself is transcribed by spRNAP-IV, suggesting a plausible transcription coupling between nucleus and mitochondria. Our results support a new mechanism of transcription of a subset of nuclear genes using the fourth RNA polymerase spRNAP-IV.

## Results

### Muscle and non-muscle actin genes show differential sensitivity to RNA polymerase II inhibitors

**Figure 1:**
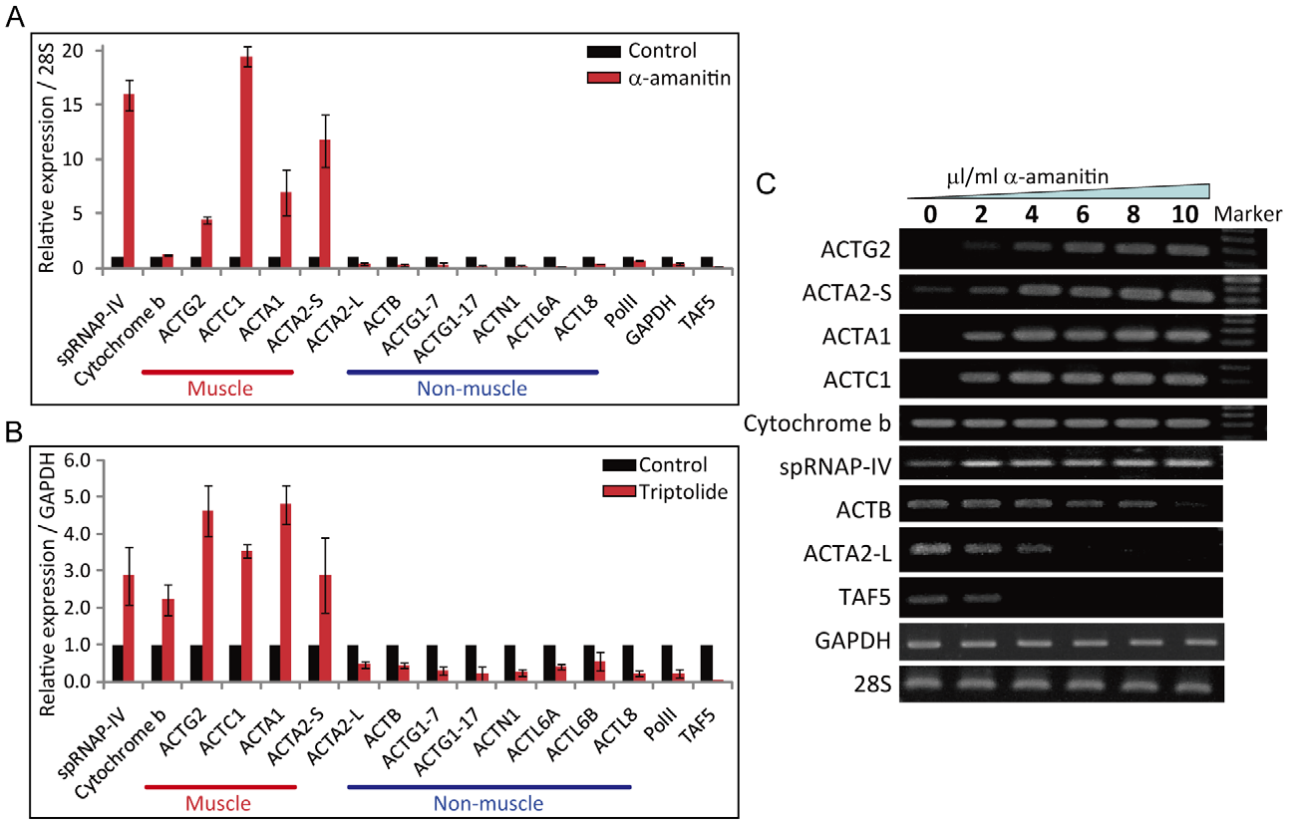
Muscle actin genes are resistant and stimulated by α-amanitin and triptolide. (A) Quantitative RT-PCR of actin genes in MCF-7 cells treated with or without 10 µg/ml of α-amanitin for 48 h. A relative expression normalized over *28s* is displayed. (N = 3, mean ± S.D.) (B) Quantitative RT-PCR of actin genes in MCF-7 cells treated with or without 0.3 µM of triptolide for 24 h. A relative expression normalized over *GAPDH* is displayed. (N = 3, mean ± S.D.) (C) RT-PCR of actin genes in MCF-7 cells treated with increasing concentration of α-amanitin for 48 h showing concentration- dependent stimulation of muscle actin transcripts.

The actin group of genes can be classified into muscle actins and non-muscle actins. The muscle actins include cardiac, smooth, and skeletal muscle actins encoded by genes *ACTC1*, *ACTG2*, *ACTA2*, and *ACTA1*. The non-muscle type actins include genes such as *ACTG1*, *ACTB*, *ACTN1*, *ACTL6A*, and *ACTL8T*. In the microarray analysis we found that muscle actin genes were resistant to PolII inhibitor α-amanitin in contrast to non-muscle actin genes which were sensitive. We then performed q-PCR analysis to confirm these results (Figure 1A; effect of different concentrations of α-amanitin is shown in S1A). The q-PCR analysis even indicated that muscle actin mRNAs were up-regulated by α-amanitin treatment whereas cytoplasmic actin genes were inhibited. As expected, PolII, transcription factor *TAF5* as well as *GAPDH* were sensitive to this drug.

We further confirmed resistance of muscle actin expression to PolII inhibition using another PolII inhibitor, triptolide (Figure 1B; effect of different concentrations of triptolide is shown in S2B). These results suggest that the two types of actin genes are transcribed by different transcription mechanisms. Interestingly, vascular smooth muscle *ACTA2* gene has two different promoters and the short form, NM_001613 (here after referred to as *ACTA2*-S) is resistant, but the long form, NM_001141945 (here after referred to as *ACTA2*-L) is sensitive to α-amanitin and triptolide in MCF-7 cell line. The resistance of muscle actin genes to triptolide treatment was also observed in MCF-10F, MBA-MD-231, MDA-MB-435s and human umbilical vein smooth muscle cells (HUVSMC) cells (Figure S1C - F). Strong induction of muscle actin genes by TNF-α was also found to be resistant to PolII inhibitors (Figure S2).

To further demonstrate insensitivity of muscle actin gene expression to α-amanitin, we analyzed the level of mRNA in cells treated with increasing amount of the drug (Figure 1C). The result clearly showed that the muscle actin genes *ACTG2*, *ACTA2*-S, *ACTC1*, and *ACTA1* were resistant or even up-regulated with increasing amount of α-amanitin, whereas the non-muscle genes, *ACTB* and *ACTA2*-L, were sensitive to the drug. The mitochondrial RNA polymerase spRNAP-IV and mitochondria encoded cytochrome b were resistant to the drug whereas *TAF5* and *GAPDH* were sensitive to α-amanitin inhibition.

### Expression of muscle actin genes was resistant to knockdown to PolII but sensitive to *POLRMT* knockdown

**Figure 2:**
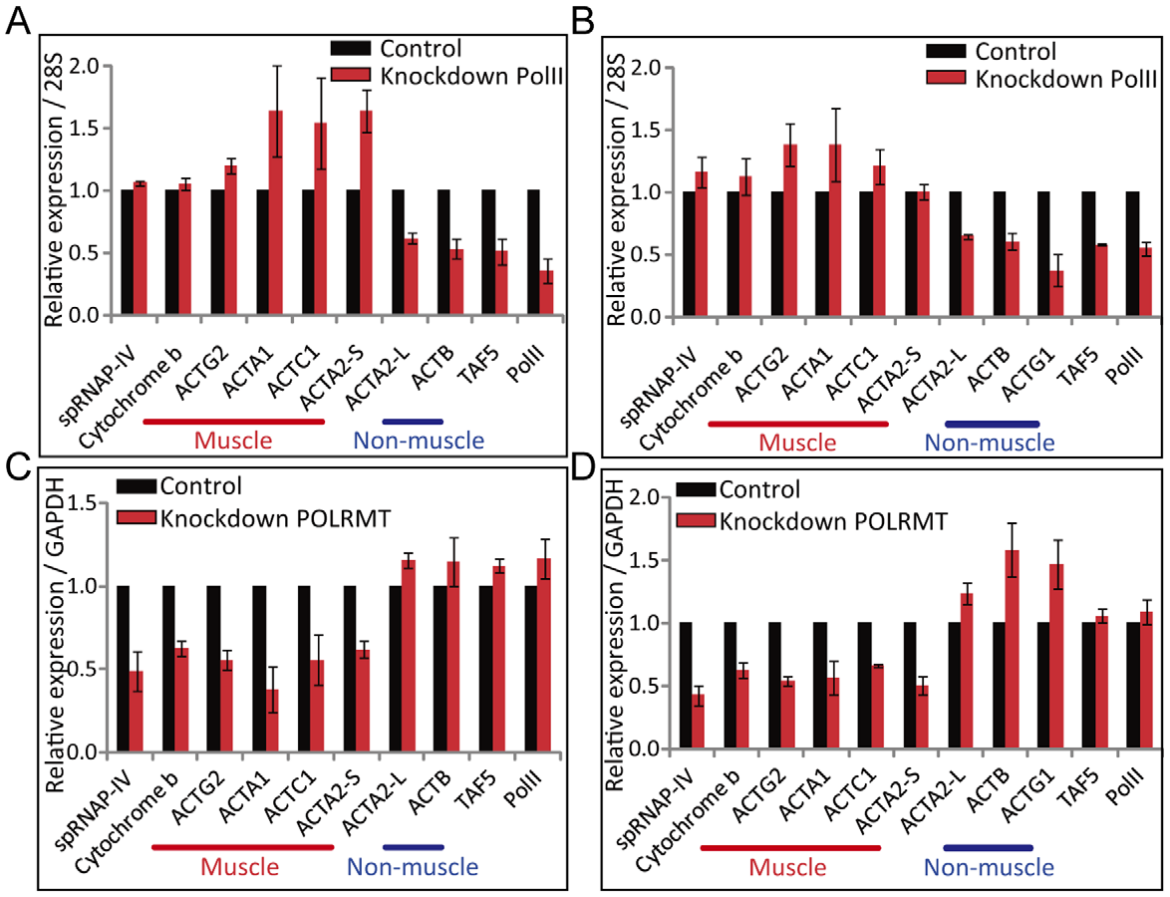
Muscle actin genes are resistant to PolII knockdown, but sensitive to *POLRMT* knockdown. Quantitative RT-PCR of actin genes in (A) MCF-7 cells and (B) HUVSMC cells subjected to siRNA knockdown of PolII for 72 h. A relative expression normalized over *28s* is displayed. (N = 3, mean ± S.D.) Quantitative RT-PCR of actin genes in (A) MCF-7 cells and (B) HUVSMC cells subjected to siRNA knockdown of *POLRMT* for 72 h. A relative expression normalized over *GAPDH* is displayed. (N = 3, mean ± S.D.)

The results described above suggest that muscle actin genes are not transcribed by PolII. To test this hypothesis, we carried out analysis of muscle and non-muscle actin mRNA 72 hours after knockdown of PolII. The results showed that muscle actin genes *ACTG2*, *ACTA2*-S, *ACTC1*, *ACTA1* as well as mitochondria cytochrome b were insensitive to PolII knockdown, whereas *ACTB*, *ACTA2*-L and *TAF5* were down-regulated after knockdown in both MCF-7 and HUVSMC cells (Figure 2A and 2B). This result further demonstrates that muscle actin genes are not transcribed by PolII. As Kravchenko *et al*., have shown that a differentially spliced mitochondrial RNA polymerase spRNAP-IV is involved in the transcription of some nuclear genes resistant to α-amanitin [1], we then tested whether muscle actin genes were transcribed by this polymerase. Indeed, muscle actin genes expression was down-regulated after *POLRMT* knockdown but not non-muscle actin genes in both MCF-7 and HUVSMC cell lines (Figure 2C and 2D). These results further support the hypothesis that muscle actin genes are transcribed by spRNAP-IV, whereas the non-muscle actin genes are transcribed by PolII.

### Nuclear run-on transcription of muscle actin genes were resistant to α-amanitin and triptolide treatments and PolII knockdown but down-regulated after spRNAP-IV knockdown

**Figure 3:**
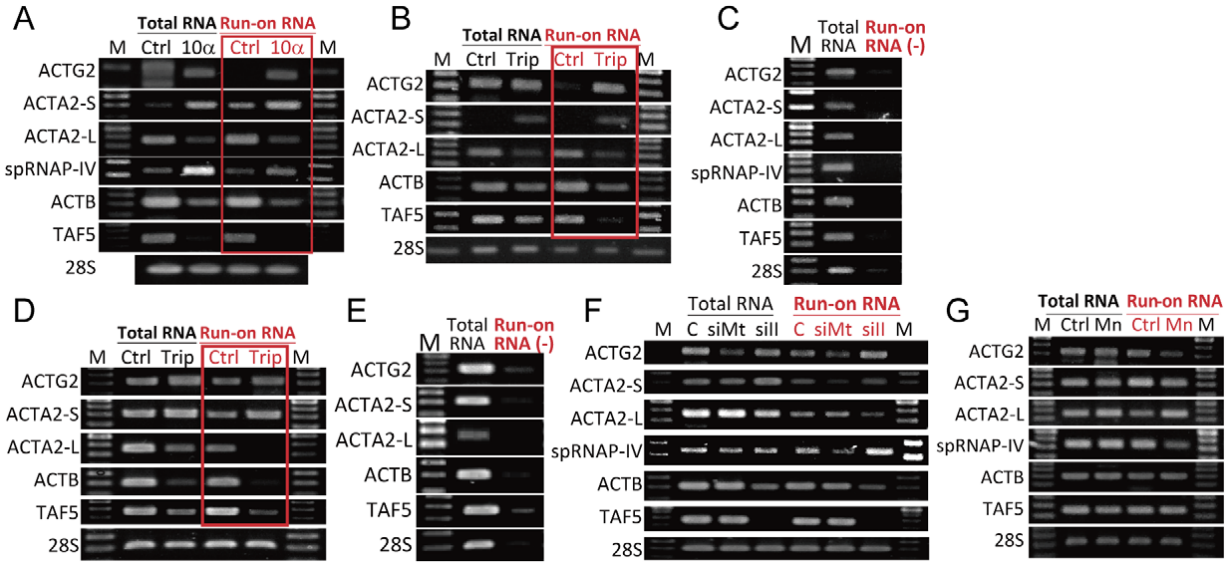
Nuclear Run-on assay of muscle actin genes transcription. (A) MCF-7 cells were treated with or without 10 µg/ml of α-amanitin for 48 h. (B) MCF-7 cells were treated with or without 0.3 µM of triptolide for 24 h (C) Run-on reaction was performed omitting biotinylated nucleotides as negative control in MCF-7 cells (D) HUVSMC cells were treated with or without 0.3 µM of triptolide for 24 h. (E) Run-on reaction was performed omitting biotinylated nucleotides as negative control in HUVSMC cells. (F) MCF-7 cells were subjected to siRNA knockdown of either non-targeting control (labeled C), PolII (labeled siII) or *POLRMT* (labeled siMt) for 72 h. (G) Nuclei were prepared and run-on reaction was performed either without (ctrl) or with the presence of 2 mM of Manganese ion (Mn). Nuclei were prepared and run-on reaction was performed by incubation with GTP, CTP, ATP, and biotin-16-UTP. Total RNA was prepared and biotinylated RNA was isolated using streptavidin magnetic beads (Run-on RNA). *ACTG2*, *ACTA2*-S, *ACTA2*-L, spRNAP-IV, *ACTB*, *TAF5*, and *28S* RNA levels in total RNA (Total RNA in black), biotinylated RNA (Run-on RNA in red), and unlabeled RNA (Run-on RNA (-) in red) were determined by RT-PCR. (M: marker; Ctrl: control; 10α: 10 µg/ml of α-amanitin; Trip: 0.3 µM of triptolide; siC: knockdown control; siMT: knockdown *POLRMT*; siII: knockdown PolII; Mn: 2 mM of Mn ion).

Since mRNA level can be affected by transcription, post-transcriptional processing or RNA stability, resistance of expression to PolII inhibitors could possibly be due to factors other than transcription. To demonstrate that inhibition indeed occurs at transcriptional level, we carried out nuclear run-on experiment after cells were treated with PolII inhibitors. The run-on RNA was labeled with biotin and the run-on products were affinity purified. The nature of the affinity-purified nascent RNA was then analyzed by RT-PCR reaction. The results showed that expression of muscle actin *ACTG2* and *ACTA2*-S as well as spRNAP-IV were indeed induced by α-amanitin and triptolide, whereas *ACTB*, *ACTA2*-L, and *TAF5* were down-regulated (Figure 3A, 3B, and 3D) in both MCF-7 and HUVSMC cells. Control experiments without biotin labeling did not show the run-on products (Figure 3C and 3E).

We also carried out nuclear run-on after knockdown of either PolII or *POLRMT*. PolII knockdown led to decreased run-on product of *ACTB* and *TAF5* but not muscle actin genes *ACTG2* and *ACTA2*-S. On the contrary, knockdown of *POLRMT* inhibited muscle actin gene transcription but not the transcription of non-muscle genes (Figure 3F).

### Presence of manganese ions inhibits *in vitro* RNA synthesis performed by spRNAP-IV

Since mitochondrial RNA polymerase is known to be inhibited by manganese whereas PolII can transcribe in the presence of this divalent ion [4], muscle actin gene transcription *in vitro* would be inhibited by manganese if spRNAP-IV is involved in the transcription of these genes. Nuclear run-on experiments indeed confirm that *ACTG2*, *ACTA2*-S and spRNAP-IV are down-regulated in the presence of manganese. In contrast, *ACTB*, *TAF5* and *ACTA2*-L, which are PolII genes, are not affected by this divalent ion (Figure 3G).

### Reporter expression driven by *ACTG2* and *ACTA2*-S promoter was resistant to triptolide treatment

**Figure 4:**
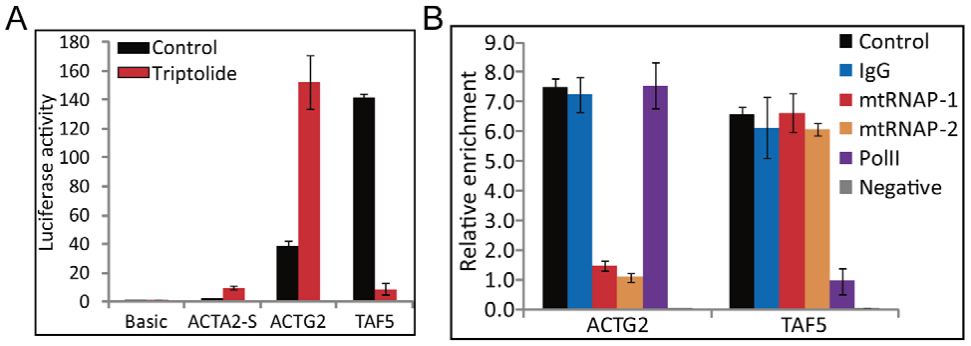
Reporter and *in vitro* transcription assays of muscle actin gene promoters. Promoter regions of *ACTG2*, *ACTA2*-S, and *TAF5* were constructed and cloned into the pGL3-basic reporter plasmid. (A) Luciferase activity assays were performed with transient transfections of pGL3-basic, pGL3-ACTA2-S-promoter, pGL3-ACTG2-promoter, and pGL3-TAF5-promoter treated with or without 0.3 µM of triptolide for 24 h. pGL3-basic was used as control and the relative fold change of luciferase activity of each gene was shown. (N = 3, mean ± S.D.) (B) pGL3-ACTA2-S-promoter and pGL3-ACTG2-promoter plasmids were digested into linear forms by NotI. *In vitro* transcription assays were performed by using the HeLaScribe^R^ Nuclear Extract *in vitro* Transcription System (Promega). Anti-RNA polymerase II (PolII), anti-mitochondrial RNA polymerase (mtRNAP-1 and mtRNAP-2) and Anti-IgG (IgG) antibodies were each pre-incubated with nuclear extract. *In vitro* transcription was performed by incubation with GTP, CTP, ATP, and biotin-16-UTP. Negative control (Negative) was performed by incubation with non biotin labeled NTPs. Total RNA was prepared and biotinylated RNA was isolated using streptavidin magnetic beads. The degree of enrichment is calculated relative to the ratio of signals obtained in the total RNA. (N = 3, mean ± s.e.m.)

We employed promoter-driven reporter assay to further demonstrate that muscle actin genes are not transcribed by PolII. The upstream promoter regions of *ACTG2*, *ACTA2*-S, and *TAF5* genes were cloned into a reporter plasmid pGL3. *ACTG2* and *ACTA2*-S promoter-driven luciferase activity showed resistant or even up-regulated by triptolide treatment whereas *TAF5* promoter showed a decrease in luciferase activity after triptolide treatment (Figure 4A). These results further support the hypothesis that muscle actin genes are transcribed not by PolII but by spRNAP-IV.

### Muscle actin gene promoter transcribed *in vitro* transcription using HeLa cell extract is sensitive to antibody to spRNAP-IV but not antibody to PolII

*In vitro* transcription of *ACTG2* and *TAF5* promoters were further assayed to demonstrate that *ACTG2* is not transcribed by PolII, but by spRNAP-IV. The pGL3 plasmids containing either upstream promoter regions of *ACTG2* and *TAF5* genes used in Luciferase assay were digested into linear forms to serve as DNA template in *in vitro* transcription reaction using HeLa cell extract. The *in vitro* transcription activity of *ACTG2* was down-regulated in the presence of antibodies to mitochondrial RNA polymerase, but not to PolII antibody (Figure 4B). In contrast, the *in vitro* transcription activity of *TAF5* was significantly down-regulated in the presence of PolII antibody, but not mitochondrial RNA polymerase antibodies. These results further support the hypothesis that muscle actin genes are transcribed not by PolII but by spRNAP-IV.

### Direct binding of spRNAP-IV to muscle actin gene promoter regions

**Figure 5:**
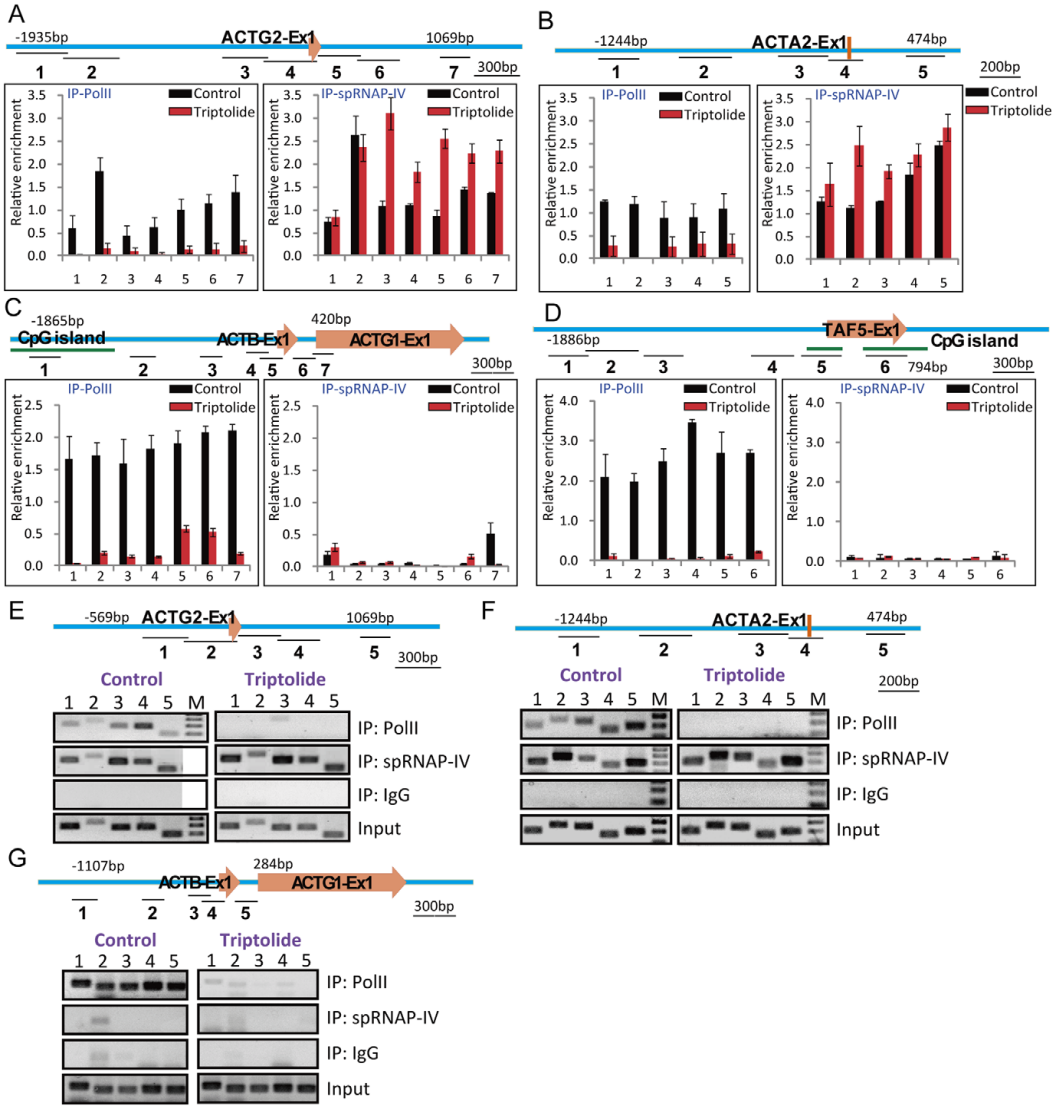
spRNAP-IV binding to muscle actin gene promoters before and after triptolide treatment by chromatin immunoprecipitation. Chromatin immunoprecipitation with anti-PolII (IP-PolII) and anti-spRNAP-IV (IP-spRNAP-IV) was performed in MCF-7 cells treated with or without 0.3 µM of triptolide for 24 h. DNA isolated from immunoprecipitated chromatin was subjected to PCR to amplify DNA fragments. Nonimmune immunoglobulin G (IgG) -immunoprecipitated DNA was used as the control. The degree of enrichment is calculated relative to the ratio of signals obtained in the input DNA fractions. Green lines indicate CpG islands. Orange arrows indicate the first exon of each gene. Significant spRNAP-IV binding was detected in MCF-7 cells for (A) *ACTG2* and (B) *ACTA2*-S promoters treated with or without triptolide. spRNAP-IV or PolII occupancy was not observed in promoters of (C) *ACTB* and (D) *TAF5* after triptolide treatment. Data are the means ± standard errors of the means (s.e.m.) from three independent experiments. Significant spRNAP-IV binding was also detected in HUVCMS cells for (E) *ACTG2* and (F) *ACTA2*-S promoters treated with or without triptolide. spRNAP-IV or PolII occupancy was not observed in promoter of (G) *ACTB* after triptolide treatment. M: Marker.

We employed chromatin immunoprecipitation (ChIP) to determine whether spRNAP-IV binds directly to the promoter regions of *ACTG2* and *ACTA2*-S. As shown in Figure 5, ChIP experiments showed that spRNAP-IV binds to promoter regions of *ACTG2* and *ACTA*2-S but not *ACTB* and *TAF5*, and that the promoter binding is insensitive to triptolide treatment in both MCF-7 and HUVSMC cell lines. Knockdown of spRNAP-IV resulted in the depletion of this polymerase from the promoter of muscle actin genes (Figure S3).

### Transcription of muscle actin genes by spRNAP-IV might provide an energy-dependent coupling between mitochondria and nuclear transcription

**Figure 6:**
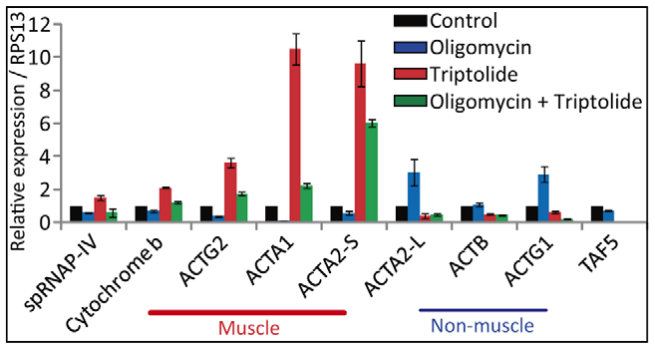
Muscle actin genes are repressed by oligomycin. Quantitative RT-PCR of actins in MCF-7 cells untreated or treated with oligomycin, triptolide, or both drugs. To determine the affect of oligomycin, cells were cultured in the presence of 10 µg/ml of oligomycin for 24 hours. Triptolide was treated to the cells for 20 hours prior RNA extraction. The basal expression of muscle actins was repressed by oligomycin. Induction of muscle actin genes by triptolide was down-regulated in the presence of oligomycin. In contrast, the expression of *ACTA2*-L and *ACTG1* non-muscle actin genes showed increased expression under the presence of oligomycin and is repressed by triptolide. Oligomycin had no significant affect on *ACTB*. A relative expression normalized over *RPS13* is displayed. (N = 3, mean ± S.D.)

To assess the biological meaning of transcription of energy-dependent contractile muscle actin genes by spRNAP-IV, we tested the possibility that muscle actin gene transcription is coupled to ATP production in the mitochondria. Inhibition of mitochondrial function by oligomycin alone repressed the basal level expression of *ACTG2*, *ACTA2*-S, *ACTA1*, *ACTC1, POLRMT,* and cytochrome b. Induced expression of muscle actins by triptolide is down-regulated by oligomycin as well (Figure 6). Inhibition of muscle actin genes and *POLRMT* is consistent with the hypothesis that transcription of muscle actin genes is coupled to energy production in the mitochondria.

## Discussion

In this report, we report that smooth, cardiac, and skeletal muscle actins are transcribed by nuclear form of mitochondrial polymerase, spRNAP-IV. This is demonstrated both by insensitivity to PolII inhibition and knockdown, sensitivity to spRNAP-IV inhibition or knockdown, as well as binding of spRNAP-IV to the promoter of muscle actin genes. As expression level can be affected by transcription, RNA processing and RNA turnover rate, we also used nuclear run-on transcription to demonstrate that muscle genes are indeed transcribed by spRNAP-IV but not by PolII. The combination of these various criteria is required to rule out RNA stability or differential processing as the cause of insensitivity to PolII inhibition. Microarray analysis alone showed many α-amanitin or triptolide resistant genes but many of them could not fulfill these criteria and therefore are not truly transcribed by spRNAP-IV. Our data and that of Kravchenko *et al*., [1] thus provide compelling evidence that some nuclear genes are transcribed not by the conventional PolII transcription machinery but by a fourth RNA polymerase, spRNAP-IV. Our results further indicate that a specific group of genes, namely, muscle actin genes, are specifically transcribed by spRNAP-IV and dependent on mitochondria ATP production.

Actin genes are evolved from an ancestral cytosplasmic prototype through gene duplication, with the muscle actin genes evolved later on [5] through amino acid substitutions, loss and acquisition of introns as well as the acquisition of different regulatory sequences for tissue-specific expression [6]. Our data suggest that muscle actin genes have acquired the promoter element for spRNAP-IV during evolution. The *cis*-regulatory elements responsible for spRNAP-IV-mediated transcription remain to be elucidated.

Interestingly, these energy-dependent contractile muscle actin genes are usually expressed at a higher level in organs with higher number of mitochondrion, such as heart and smooth muscle. Transcription of these genes by mitochondrial polymerase nuclear isoform might provide an energy-dependent coupling between mitochondria and nuclear transcription program for such tissues. It is possible that the splicing reaction for producing spRNAP-IV is the critical event in mitochondria-nucleus coupling. Further analysis is required to assess the biological meaning of transcription of nuclear genes by a truncated form of a mitochondrial enzyme.

It is interesting that muscle actin genes and spRNAP-IV are actually up-regulated by PolII inhibitors. Similar finding for *ZBTB1*, MGC3265 and *ALDH12* was observed by Kravchenko *et al*., [1]. The up-regulation of muscle actin genes could be due to the de-regulation of *POLRMT* gene after triptolide or α-amanitin treatment. Examination of *POLRMT* gene shows that its transcription converges with *HCN2* gene, which is transcribed by PolII. Inhibition of *HCN2* transcription may thus relieve the interference of the converging transcription. The presence of some PolII in the 5′ region of muscle actin genes may represent the interfering PolII transcription, and inhibition of PolII thus could also account for the up-regulation of muscle actins. We believe that the PolII found in the 5′ region of muscle actin genes does not participate in transcription of these genes, since knockdown of PolII did not affect their transcription *in vitro* or expression level *in vivo*. Our preliminary results suggest that several muscle actin genes may have antisense transcription in or near these genes, and thus inhibition of PolII could relieve the interference of these antisense transcriptions on muscle actin gene expression.

The biological significance of transcription of nuclear genes by a truncated form of mitochondrial polymerase is still unclear at present. Mitochondria-nucleus communication and crosstalk has been shown from yeast to plants and mammalian cells, [7] and retrograde regulation of nuclear genes by mitochondria has been well documented [8], [9]. Genes transcribed by spRNAP-IV might participate in such crosstalk. It is interesting that all the muscle actin genes contain in their promoters SRF binding site, which has been found in the promoters of genes involved in mitochondria biogenesis such as PGC-1α and muscle contraction [10].

It is interesting that our data show that *POLRMT*, which encodes mitochondrial RNA polymerase, is itself transcribed by spRNAP-IV, forming a plausible feedback loop. Since Kravchenko *et al*., have shown that full-length mitochondria RNA polymerase is not present in the nucleus [1], nuclear isoform of mitochondria (spRNAP-IV) is the most likely candidate that serves as a factor for nucleus-mitochondria communication and coupling through differential splicing of the primary transcript of *POLRMT*.

## Materials and Methods

### Cell culture and Treatments

MCF-7, MDA-MB-231, MDA-MB-435s, MCF-10F cell lines were originally obtained from ATCC (Manassas, VA). MCF-7 cells were cultured in RPMI medium supplemented with 10% fetal bovine serum. MDA-MB-231 and MDA-MB-435s cells were cultured in DMEM medium supplemented with 10% fetal bovine serum. MCF-10F cells were cultured in a volume of 1∶1 mixture of DMEM and F12 medium, supplemented with 20 ng/ml epidermal growth factor, 100 ng/ml cholera toxin, 0.01 mg/ml insulin, 500 ng/ml hydrocortisone, and 5% horse serum (Sigma). All cell lines were incubated in a humidified 37°C incubator with 5% CO_2_. HUVSMC cell line was originally obtained from Cell Applications, Inc. HUVSMC cells were routinely passaged and maintained in smooth muscle cell growth medium (Cell Applications, Inc.). To determine the inhibition of PolII transcription, cells were cultured in the presence of 10 µg/ml of α-amanitin or 0.3 µM of triptolide (Sigma) for 48 or 24 h, respectively.

### RNA extraction and Affymetrix

Total RNA was extracted according to the RNeasy® Mini Kit Spin Protocol (QIAGEN). Affymetrix microarray was performed using Human U133 plus 2.0 (Affymetrix). Details of the methods for RNA quality, sample labeling, hybridization and expression analysis were according to the manual of Affymetrix Microarray Kit.

### Microarray

All Affymetrix data is MIAME compliant and that the raw data has been deposited in a MIAME compliant database, GEO. The microarray data were deposited at the NCBI GEO website (GEO accession number GSE30350).

### Reverse Transcription and Quantitative Real-Time PCR

Reverse transcription was performed by using superScript™ III RNase H - Reverse Transcriptase (Invitrogen). Total cDNA was then synthesized by means of Oligo(dT)_15_ or random hexamer primed reverse transcription of captured molecules. Quantitative PCR (q-PCR) was performed using SYBR Green dye on Roche Applied Science LightCycler® 2.0 Real-Time PCR System. All reactions were performed in triplicate with SYBR Green Master Mix (Sigma) plus 20 µM of both the forward and reverse primer according to the manufacturer's recommended thermocycling conditions, and then subjected to melting curve analysis. The calculated quantity of the target gene for each sample was divided by the average sample quantity of the housekeeping genes, glyceraldehydes-3-phosphate dehydrogenase (*GAPDH*) or *28S* to obtain the relative gene expression.

### Run-on assay

Nuclear run-on reactions were performed by supplying biotin-16-UTP to nuclei, and labeled transcripts were bound to streptavidin-coated magnetic beads as described by Patrone G. *et al*. 2000 [11] with minor modifications. Nuclei were prepared from MCF-7 cells by resuspension in Nonidet P-40 lysis buffer (10 mM HEPES, pH 7.3, 10 mM NaCl, 3 mM MgCl_2_, 150 mM sucrose, and 0.5% Nonidet P-40). Nuclei were isolated, and the pellets were resuspended in 1 ml of glycerol buffer (50 mM Tris-Cl, pH 8.3, 40% glycerol, 5 mM MgCl_2_, and 0.1 mM EDTA). 1 ml of transcription buffer (20 mM Tris-Cl, pH 8.0, 200 mM KCl, 5 mM MgCl_2_, 4 mM dithiothreitol, 4 mM each of ATP, GTP, and CTP, 200 mM sucrose, and 20% glycerol) was added in the nuclei along with 10 µl of biotin-16-UTP or UTP for run-on reaction or negative control, respectively (Roche Diagnostics). After incubation at 29°C for 30 min, the reaction was terminated by the addition of 6 µl of 250 mM CaCl_2_, and 6 µl of RNase-free DNase I and incubated at 29°C for 10 min. To purify RNA, a TRIreagent extraction, phenol-chloroform extraction, and isopropanol (Sigma) precipitation were then performed. A small aliquot (5 µl from a total of 50 µl) was saved as “total nuclear RNA” for each treatment. Dynabeads M-280 streptavidin (Dynal Biotech) were mixed with an equal volume of the isolated RNA samples for 20 min at 42°C for 20 min and 2 h at room temperature. After washing with 15% formamide and 2X SSC, the beads were resuspended in 45 µl of nuclease-free water. Reverse transcription was performed by using superscript™ III RNase H - Reverse Transcriptase (Invitrogen). Total cDNA was then synthesized by means of random hexamer primed reverse transcription of captured molecules.

### SiRNA knockdown assay

SiRNA knockdown assay was performed according to the manufacturer's protocol (pSUPER RNAi System, OligoEngine) [12]. Inhibition of expression of PolII or *POLRMT* was achieved by transfection with pSUPER vector constructs expressing hairpin siRNAs controlled by H1 RNA gene promoter. The following 19 bp regions corresponding to appropriate mRNAs were present in the hairpin transcripts: exon 5 of *POLRMT* gene, 5′ – GATCCCCCACCTCCAAGCTGCTCAGGTTCAAGAGACCTGAGCAGCTTGGAGGTGTTTTTA – 3′ and 5′–AGCTTAAAAACACCTCCAAGCTGCTCAGGTCTCTTGAACCTGAGCAGCTTGGAGGTGGGG – 3′; exon 17 of *POLRMT* gene, 5′ – GATCCCCCTTCATCCACTCGCTGGACTTCAAGAGAGTCCAGCGAGTGGATGAAGTTTTTA – 3′ and 5′ – AGCTTAAAAACTTCATCCACTCGCTGGACTCTCTTGAAGTCCAGCGAGTGGATGAAGGGG – 3′; PolII large subunit (*POLR2A*), 5′ – GATCCCCCCTCTCCAAGCTACTCTCCTTCAAGAGAGGAGAGTAGCTTGGAGAGGTTTTTA – 3′ and 5′ – AGCTTAAAAACCTCTCCAAGCTACTCTCCTCTCTTGAAGGAGAGTAGCTTGGAGAGGGGG – 3′. The purification of plasmid DNA was performed according to the QIAprep® Spin Miniprep Kit Instruction Manual (QIAGEN). Cells were transfected using Lipofectamine™ LTX according to the instructions of the manufacturer (Invitrogen). Cells were transfected in serum-free medium. After 20 h, the siRNA containing medium was replaced with complete medium.

### Chromatin Immunoprecipitation (ChIP)

ChIP assay was performed according to the manufacturer's protocol (Upstate Biotechnology, Inc., Lake Placid, NY) except that sonication condition was changed to 45 times for 1 secs each at 3W output. Human MCF-7 cells were fixed for 10 min with 1% formaldehyde. The cells were lysed and the chromatin sonicated to 200–1000 bp fragments. Chromatin was immunoprecipitated by using mtRNAP/spRNAP-IV (ab93102, abcam) or RNA polymerase II antibody (ab5131, abcam), and Protein A Agarose beads. The beads were washed once with each washing buffer, including low salt immune complex wash buffer, high salt immune complex wash buffer, and LiCl immune complex wash buffer, and twice with 1X TE buffer. Precipitates were eluted with 1% of SDS and 100 mM of NaHCO_3_. The samples were heated at 65°C for 6 h in order to reverse cross-link, extracted with phenol/chloroform, ethanol-precipitated. Then PCR amplification was performed.

### Luciferase assay

The promoter regions of *ACTG2*, *ACTA2*-S, and *TAF5* were cloned into the pGL3-basic Reporter Vector (Promega). MCF-7 cells were transfected using Lipofectamine™ LTX according to the instructions of the manufacturer (Invitrogen). The plasmids were transfected for 6 h and then treated with 0.3 µM of triptolide for 24 h. Post 30 h of incubation, the cells were lysed and luciferase assay was conducted using the Luciferase Assay System (Promega) using a fluorimetric plate reader.

### *In vitro* Transcription System

*In vitro* transcription was performed by using HeLaScribe^R^ Nuclear Extract *in vitro* Transcription System (Promega Cat.# E3110) according to the manufacturer's protocol with slight modifications. Anti-RNA polymerase II [PolII (ab5131, abcam)], anti-mitochondrial RNA polymerase [mtRNAP-1 (ab32954, abcam) and mtRNAP-2 (ab93102, abcam)] and anti-IgG (IgG) antibodies were each pre-incubated with nuclear extract and 30 µg of yeast tRNA for 15 min. *In vitro* transcription was performed by incubation with both linear forms of pGL3 plasmids containing either upstream promoter regions of *ACTG2* or *TAF5* genes used in Luciferase assay, and GTP, CTP, ATP, and biotin-16-UTP (Roche Diagnostics). Negative control was performed by incubation with non biotin labeled NTPs. After incubation at 30°C for 1 h, the reaction was terminated by the addition of 175 µl of HeLa Extract Stop Solution (Promega Cat.# E3110). To purify RNA, a TRIreagent extraction, phenol-chloroform extraction, and isopropanol (Sigma) precipitation were then performed. A small aliquot (2 µl from a total of 22 µl) was saved as “total nuclear RNA” for each condition. The biotinylated RNA was isolated using streptavidin-coated magnetic beads as described in Run-on assay. Reverse transcription was performed by using superscript™ III RNase H - Reverse Transcriptase (Invitrogen). Total cDNA was then synthesized by means of random hexamer primed reverse transcription of captured molecules.

## Supporting Information

Figure S1 **Muscle actin genes are resistant and stimulated by α-amanitin and triptolide.** (A) Quantitative RT-PCR of actin genes in MCF-7 cells treated with increasing concentrations of α-amanitin for 48 h. A relative expression normalized over *28s* is displayed. (N = 3, mean ± S.D.) (B) Quantitative RT-PCR of actin genes in MCF-7 cells treated with increasing concentrations of triptolide for 24 h. A relative expression normalized over *GAPDH* is displayed. (N = 3, mean ± S.D.) Quantitative RT-PCR of actin genes in (C) MCF-10F cells (D) MDA-MB-231 cells (E) MDA-MB-435s cells (F) HUVSMC cells treated with or without 0.3 µM of triptolide for 24 hours. The experiment was performed three independent times and a relative expression normalized over *GAPDH* is displayed. (N = 3, mean ± S.D.)
(TIF)

Figure S2 **Strong induction of muscle actin genes by TNF-α was resistant to PolII inhibitor.** Quantitative RT-PCR of actin genes in MCF-7 cells untreated or treated with TNF-α, triptolide, or both drugs. To determine the inhibition of PolII transcription, cells were cultured in the presence of 0.3 µM of triptolide for 24 hours. TNF-α was treated to the cells for 2 hours prior RNA extraction. Muscle actin genes were strongly up-regulated under TNF-α, triptolide, and both drug treatments. Induction of muscle actin genes by TNF-α was resistant to triptolide. In contrast, non-muscle actin genes, PolII and *TAF5* showed decreased expression under the presence of triptolide. A relative expression normalized over *GAPDH* is displayed. (N = 3, mean ± S.D.)
(TIF)

Figure S3 **Knockdown of** ***POLRMT*** **resulted in the depletion of this polymerase from the promoter of** ***ACTA2*** **-S.** Chromatin immunoprecipitation with anti-spRNAP-IV (IP-spRNAP-IV) was performed in MCF-7 cells transient transfected with pSUPER-control (Si-control) or knockdown of *POLRMT* (Si-POLRMT) plasmid. DNA isolated from immunoprecipitated chromatin was subjected to PCR to amplify DNA fragments. Nonimmune immunoglobulin G (IgG)-immunoprecipitated DNA was used as the control. Significant spRNAP-IV binding was detected in MCF-7 cells for *ACTA2*-S promoter in knockdown control, but knockdown of spRNAP-IV resulted in the depletion of this polymerase from the promoter. M: Marker.
(TIF)

## References

[1] Kravchenko JE, Rogozin IB, Koonin EV, Chumakov PM (2005). Transcription of mammalian messenger RNAs by a nuclear RNA polymerase of mitochondrial origin.. Nature.

[2] Kedinger C, Gniazdowski M, Mandel JL, Gissinger F, Chambon P (1970). Alpha-amanitin: a specific inhibitor of one of two DNA-pendent RNA polymerase activities from calf thymus.. Biochem Biophys Res Commun.

[3] Vispe S, DeVries L, Creancier L, Besse J, Breand S (2009). Triptolide is an inhibitor of RNA polymerase I and II-dependent transcription leading predominantly to down-regulation of short-lived mRNA.. Mol Cancer Ther.

[4] Shuey DJ, Attardi G (1985). Characterization of an RNA polymerase activity from HeLa cell mitochondria, which initiates transcription at the heavy strand rRNA promoter and the light strand promoter in human mitochondrial DNA.. J Biol Chem.

[5] Mounier N, Gouy M, Mouchiroud D, Prudhomme JC (1992). Insect muscle actins differ distinctly from invertebrate and vertebrate cytoplasmic actins.. J Mol Evol.

[6] Liu T, Wu J, He F (2000). Evolution of cis-acting elements in 5′ flanking regions of vertebrate actin genes.. J Mol Evol.

[7] Ryan MT, Hoogenraad NJ (2007). Mitochondrial-nuclear communications.. Annu Rev Biochem.

[8] Liu Z, Butow RA (2006). Mitochondrial retrograde signaling.. Annu Rev Genet.

[9] Yang J, Zhang M, Yu J (2008). Mitochondrial retrograde regulation tuning fork in nuclear genes expressions of higher plants.. J Genet Genomics.

[10] Irrcher I, Hood DA (2004). Regulation of Egr-1, SRF, and Sp1 mRNA expression in contracting skeletal muscle cells.. J Appl Physiol.

[11] Patrone G, Puppo F, Cusano R, Scaranari M, Ceccherini I (2000). Nuclear run-on assay using biotin labeling, magnetic bead capture and analysis by fluorescence-based RT-PCR.. Biotechniques.

[12] Brummelkamp TR, Bernards R, Agami R (2002). A system for stable expression of short interfering RNAs in mammalian cells.. Science.

