# Supplementary figures and images for: Transcription of Muscle Actin Genes by a Nuclear Form of Mitochondrial RNA Polymerase
Source: PLoS One. 2011 Jul 25;6(7):e22583. doi: 10.1371/journal.pone.0022583 (PMC3143168; doi:10.1371/journal.pone.0022583)

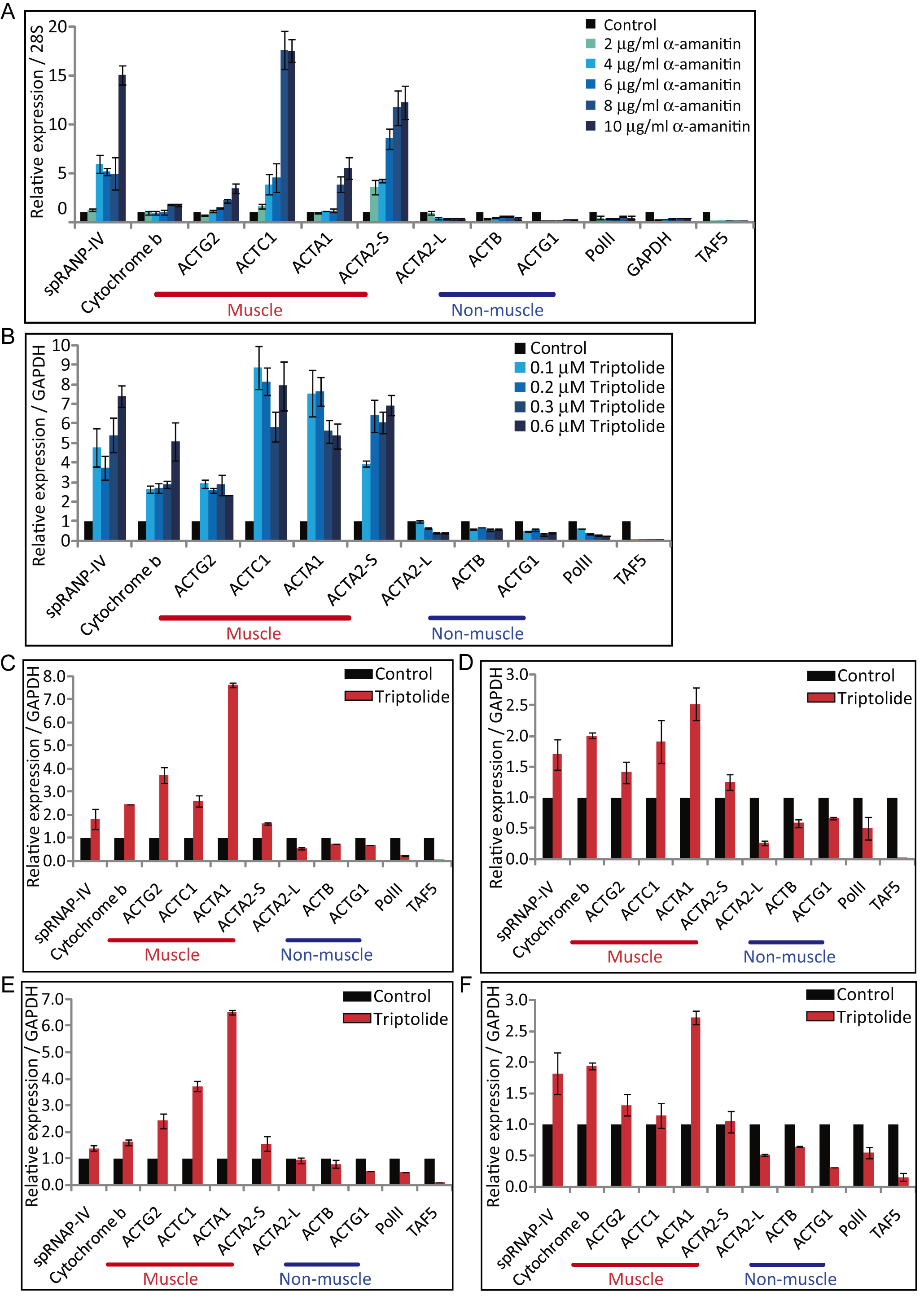

Supplement: Figure S1 — Muscle actin genes are resistant and stimulated by α-amanitin and triptolide. (A) Quantitative RT-PCR of actin genes in MCF-7 cells treated with increasing concentrations of α-amanitin for 48 h. A relative expression normalized over 28s is displayed. (N = 3, mean ± S.D.) (B) Quantitative RT-PCR of actin genes in MCF-7 cells treated with increasing concentrations of triptolide for 24 h. A relative expression normalized over GAPDH is displayed. (N = 3, mean ± S.D.) Quantitative RT-PCR of actin genes in (C) MCF-10F cells (D) MDA-MB-231 cells (E) MDA-MB-435s cells (F) HUVSMC cells treated with or without 0.3 µM of triptolide for 24 hours. The experiment was performed three independent times and a relative expression normalized over GAPDH is displayed. (N = 3, mean ± S.D.) (TIF) [file pone.0022583.s001.tif]

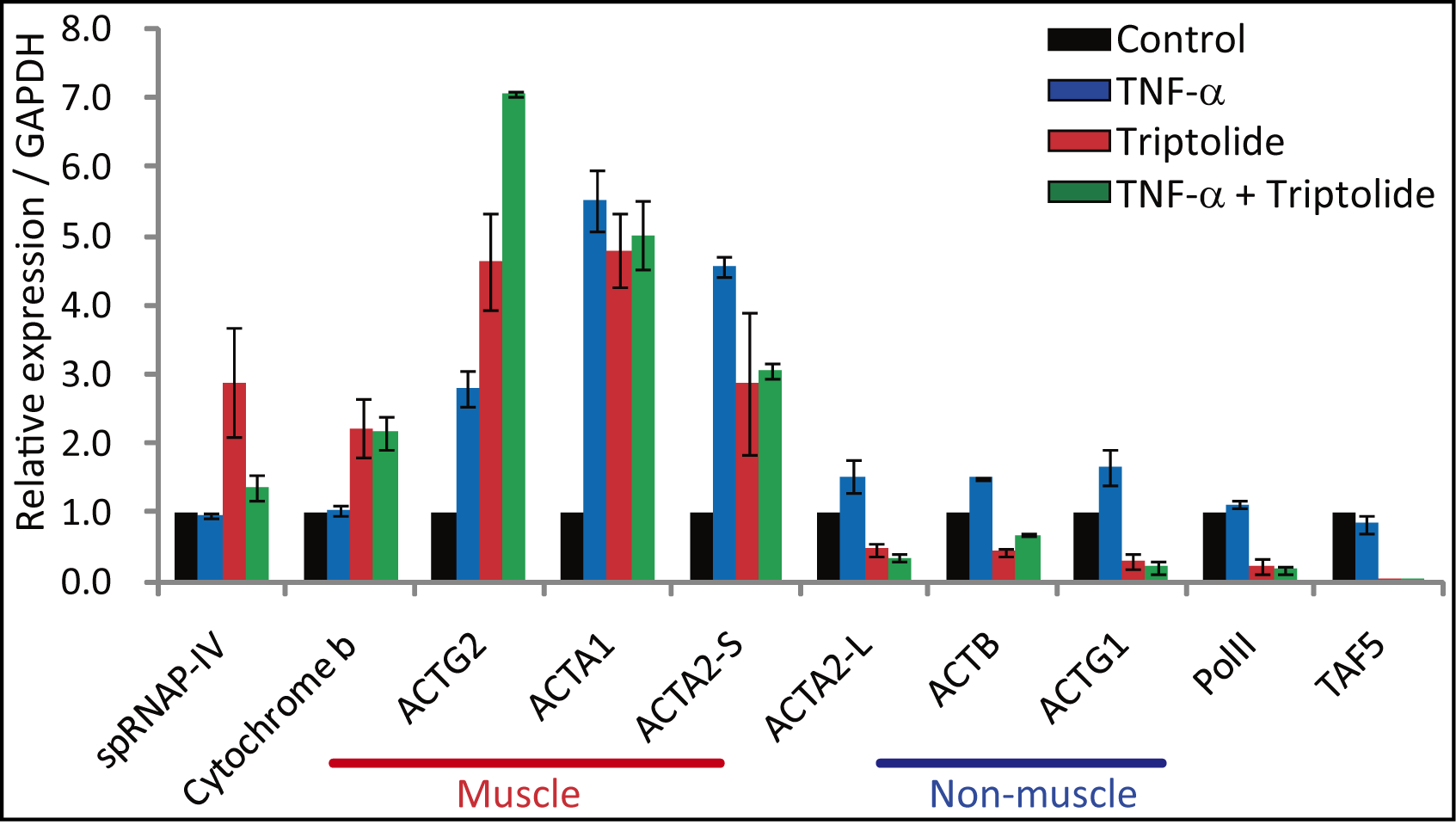

Supplement: Figure S2 — Strong induction of muscle actin genes by TNF-α was resistant to PolII inhibitor. Quantitative RT-PCR of actin genes in MCF-7 cells untreated or treated with TNF-α, triptolide, or both drugs. To determine the inhibition of PolII transcription, cells were cultured in the presence of 0.3 µM of triptolide for 24 hours. TNF-α was treated to the cells for 2 hours prior RNA extraction. Muscle actin genes were strongly up-regulated under TNF-α, triptolide, and both drug treatments. Induction of muscle actin genes by TNF-α was resistant to triptolide. In contrast, non-muscle actin genes, PolII and TAF5 showed decreased expression under the presence of triptolide. A relative expression normalized over GAPDH is displayed. (N = 3, mean ± S.D.) (TIF) [file pone.0022583.s002.tif]

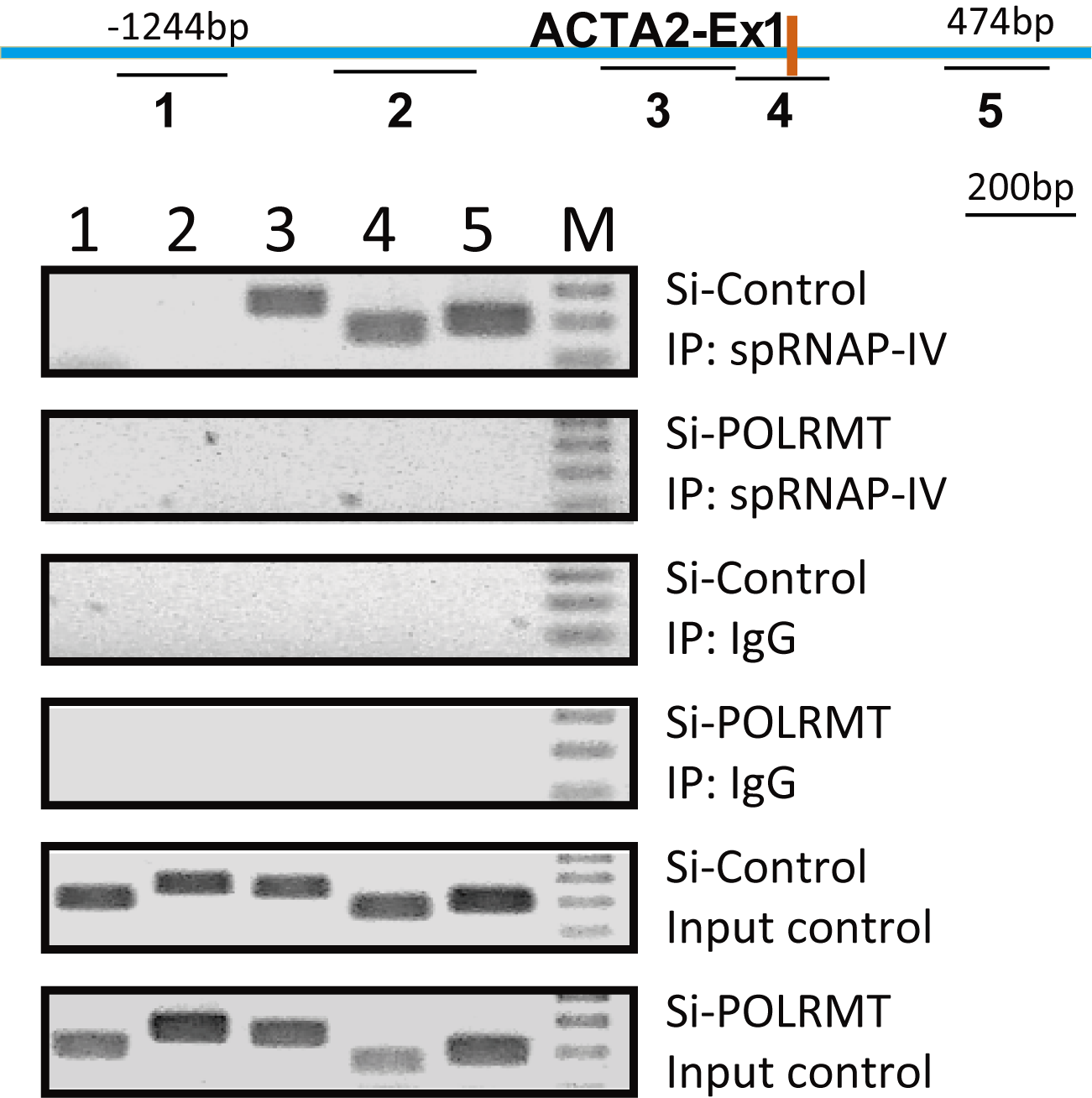

Supplement: Figure S3 — Knockdown of POLRMT resulted in the depletion of this polymerase from the promoter of ACTA2 -S. Chromatin immunoprecipitation with anti-spRNAP-IV (IP-spRNAP-IV) was performed in MCF-7 cells transient transfected with pSUPER-control (Si-control) or knockdown of POLRMT (Si-POLRMT) plasmid. DNA isolated from immunoprecipitated chromatin was subjected to PCR to amplify DNA fragments. Nonimmune immunoglobulin G (IgG)-immunoprecipitated DNA was used as the control. Significant spRNAP-IV binding was detected in MCF-7 cells for ACTA2-S promoter in knockdown control, but knockdown of spRNAP-IV resulted in the depletion of this polymerase from the promoter. M: Marker. (TIF) [file pone.0022583.s003.tif]
